# Diagnostic performance and comparison of ultrasensitive and conventional rapid diagnostic test, thick blood smear and quantitative PCR for detection of low-density *Plasmodium falciparum* infections during a controlled human malaria infection study in Equatorial Guinea

**DOI:** 10.1186/s12936-022-04103-y

**Published:** 2022-03-24

**Authors:** Maxmillian Mpina, Thomas C. Stabler, Tobias Schindler, Jose Raso, Anna Deal, Ludmila Acuche Pupu, Elizabeth Nyakarungu, Maria del Carmen Ovono Davis, Vicente Urbano, Ali Mtoro, Ali Hamad, Maria Silvia A. Lopez, Beltran Pasialo, Marta Alene Owono Eyang, Matilde Riloha Rivas, Carlos Cortes Falla, Guillermo A. García, Juan Carlos Momo, Raul Chuquiyauri, Elizabeth Saverino, L. W. Preston Church, B. Kim lee Sim, Bonifacio Manguire, Marcel Tanner, Carl Maas, Salim Abdulla, Peter F. Billingsley, Stephen L. Hoffman, Said Jongo, Thomas L. Richie, Claudia A. Daubenberger

**Affiliations:** 1 Swiss Tropical and Public Health Institute, Basel, Switzerland; 2 University of Basel, Basel, Switzerland; 3 Ifakara Health Institute, Ifakara, Tanzania; 4 Medical Care Development International, Malabo, Equatorial Guinea; 5 Equatorial Guinea Ministry of Health and Social Welfare, Malabo, Equatorial Guinea; 6 Medical Care Development International, Silver Spring, MD USA; 7 Sanaria Inc., 9800 Medical Center Drive, Rockville, MD 20850 USA; 8 Marathon EG production Ltd., Houston, USA

**Keywords:** Malaria, Rapid diagnostic test, Controlled human malaria infection, Thick blood smear, Low parasite density infections, Malaria pre-exposure

## Abstract

**Background:**

Progress towards malaria elimination has stagnated, partly because infections persisting at low parasite densities comprise a large reservoir contributing to ongoing malaria transmission and are difficult to detect. This study compared the performance of an ultrasensitive rapid diagnostic test (uRDT) designed to detect low density infections to a conventional RDT (cRDT), expert microscopy using Giemsa-stained thick blood smears (TBS), and quantitative polymerase chain reaction (qPCR) during a controlled human malaria infection (CHMI) study conducted in malaria exposed adults (NCT03590340).

**Methods:**

Blood samples were collected from healthy Equatoguineans aged 18–35 years beginning on day 8 after CHMI with 3.2 × 10^3^ cryopreserved, infectious *Plasmodium falciparum* sporozoites (PfSPZ Challenge, strain NF54) administered by direct venous inoculation. qPCR (18s ribosomal DNA), uRDT (Alere™ Malaria Ag P.f.), cRDT [Carestart Malaria Pf/PAN (PfHRP2/pLDH)], and TBS were performed daily until the volunteer became TBS positive and treatment was administered. qPCR was the reference for the presence of *Plasmodium falciparum* parasites.

**Results:**

279 samples were collected from 24 participants; 123 were positive by qPCR. TBS detected 24/123 (19.5% sensitivity [95% CI 13.1–27.8%]), uRDT 21/123 (17.1% sensitivity [95% CI 11.1–25.1%]), cRDT 10/123 (8.1% sensitivity [95% CI 4.2–14.8%]); all were 100% specific and did not detect any positive samples not detected by qPCR. TBS and uRDT were more sensitive than cRDT (TBS vs. cRDT p = 0.015; uRDT vs. cRDT p = 0.053), detecting parasitaemias as low as 3.7 parasites/µL (p/µL) (TBS and uRDT) compared to 5.6 p/µL (cRDT) based on TBS density measurements. TBS, uRDT and cRDT did not detect any of the 70/123 samples positive by qPCR below 5.86 p/µL, the qPCR density corresponding to 3.7 p/µL by TBS. The median prepatent periods in days (ranges) were 14.5 (10–20), 18.0 (15–28), 18.0 (15–20) and 18.0 (16–24) for qPCR, TBS, uRDT and cRDT, respectively; qPCR detected parasitaemia significantly earlier (3.5 days) than the other tests.

**Conclusions:**

TBS and uRDT had similar sensitivities, both were more sensitive than cRDT, and neither matched qPCR for detecting low density parasitaemia. uRDT could be considered an alternative to TBS in selected applications, such as CHMI or field diagnosis, where qualitative, dichotomous results for malaria infection might be sufficient.

**Supplementary Information:**

The online version contains supplementary material available at 10.1186/s12936-022-04103-y.

## Background

Over the past decade, malaria treatment and vector control interventions have significantly decreased malaria burden worldwide. The global incidence rate of malaria decreased between 2010 and 2018 from 71 to 57 cases per 1000 people at risk [1]. However, during 2020, the first full year of the COVID-19 pandemic, the World Health Organization (WHO) World Malaria Report documented an increment of 14 million annual malaria cases and 69,000 additional deaths compared to 2019, much of this attributable to COVID-19-related interruption of malaria control and medical services [2]. A significant challenge faced by malaria control and elimination projects is addressing transmission potential from low parasite density carriers with mild or no symptoms. Low parasite density carriers are estimated to account for 20–50% of human-to-mosquito transmission [3]. It would be beneficial to have a rapid test able to identify these infections.

Current widely deployed diagnostic tools such as conventional rapid diagnostic tests (cRDTs) are affordable and have user-friendly formats and function. In 2018, 259 million cRDTs were distributed mainly in sub-Saharan Africa and utilized to examine suspected malaria cases [1]. cRDTs test for the presence of histidine-rich protein 2 (PfHRP2), an antigen specific to *Plasmodium falciparum*, and many iterations of the test also include a pan-malaria antigen (PAN) common to all 4 major malaria species such as lactate dehydrogenase (LDH). While cRDTs are affordable, provide quick and readable results, and require little training to operate, they cannot detect low-density infections, which can occur frequently in low transmission areas [3–5]. As a result, efforts are underway to develop advanced malaria rapid diagnostic tests that are more sensitive and effective at identifying low-density *P. falciparum* infections [6, 7].

Laboratory-based techniques such as thick blood smear (TBS) and polymerase chain reaction (PCR) are considered to have greater sensitivity than cRDTs [8]. Historically, TBS has been the gold standard for malaria diagnosis. When performed by expert microscopists reading 0.5 µL of blood, detection may range typically between 10 and 50 parasites per microlitre (p/µL), and under carefully controlled CHMI and TBS preparation conditions, expert microscopists can quantify parasite densities at the theoretical lower limit of detection for this blood volume, 2 p/µL. In contrast, cRDTs have reported detection limits of 100–200 p/µL of blood in field studies [9–11]. TBS has the additional advantages of diagnosing infections even in the presence of parasites carrying PfHRP2 deletions, a challenge that RDT manufacturers are currently facing [12, 13], and allowing detection of all species of malaria parasite. In recent years, the further development of the quantitative polymerase chain reaction (qPCR) method has enabled the detection of low parasite densities that frequently go undetected by cRDTs and TBS [14, 15]. Utilizing qPCR, reservoirs of low parasite density cases can be identified and treated, which is an essential component for elimination in low transmission areas [8, 16–18]. The drawback of both TBS and qPCR is the need for specialized laboratory equipment, materials and well-trained staff, which are often in short supply in low income countries, making them unfeasible for use under field conditions on a large scale [19, 20]. TBS can also lead to false negative results when performed by a non-competent microscopist, the parasitaemia is low or there are multiple-species co-infections [21, 22]. An ideal diagnostic tool would combine the sensitivity of qPCR with the affordability and simplicity of the cRDT.

An ultrasensitive RDT (uRDT; Alere™ Malaria Ag P.f.) has recently been developed and offered as a reliable diagnostic tool that can be used in clinical studies and in field operations [23–25]. Similar to conventional RTDs [cRDTs, such as Carestart Malaria Pf/PAN (PfHRP2/pLDH)] in form and function, the uRDT detects PfHRP2 in *P. falciparum*, but at greater sensitivity, identifying densities as low as 0.1–1.0 p/µL in culture-derived samples [7, 23]. This more sensitive RDT, which has been described as being capable of detecting low parasite density infections, could target these low-density infections for treatment [26]. If the sensitivity reported is confirmed, it could also be used in clinical trials of anti-malarial drugs or vaccines to document protection following CHMI or natural *P. falciparum* exposure [27–29]. To date, limited literature exists systematically examining the performance of the uRDT in direct comparison to cRDTs, TBS and qPCR in samples collected from individuals with low parasite density [30, 31]. Existing investigations have been conducted predominantly in the field where it is difficult to monitor factors such as timings of infectious mosquito bites and the waxing and waning of parasite densities [24, 31–35].

A new approach to conducting controlled human malaria infection (CHMI) using *P. falciparum* sporozoites (PfSPZ) has become available over the past five years, based on administering aseptic, purified, cryopreserved, infectious PfSPZ (Sanaria® PfSPZ Challenge) [36]. In this model, PfSPZ are administered by syringe, replacing mosquito bite administration, and the study subjects are then monitored in the standard way [37]. The advantages are that CHMI can be performed by institutions without an insectary or without the need to import infected mosquitoes, the dose of PfSPZ can be standardized, and CHMI can be administered at any time without coordinated mosquito infections. The use of cryopreserved infectious PfSPZ is thus similar to the use of cryostabilates for induced blood stage malaria (IBSM), a controlled human infection model that bypasses the SPZ and liver stages and is similarly free of constraints [38]. CHMI using PfSPZ Challenge is now being utilized extensively to evaluate efficacy of anti-malarial drugs and vaccines in malaria-naive and malaria pre-exposed populations [39–45].

CHMI using injectable PfSPZ provides an opportunity to assess malaria diagnostics under carefully controlled conditions in malaria exposed populations. The exact exposure time is known, the induced infections gradually increase in density, and the time of first detection and associated prepatent period can be precisely determined. The aim of this study was to systematically evaluate and compare uRDT (Alere™ Malaria Ag P.f.) performance against three other commonly used malaria diagnostic tools using whole blood samples collected daily from malaria pre-exposed individuals undergoing CHMI.

## Methods

### Study site

The Bioko Island Malaria Elimination Program (BIMEP) focuses on developing malaria vaccines and other interventions to decrease malaria-attributable morbidity and mortality on Bioko Island, Equatorial Guinea [46]. BIMEP performs various activities on Bioko such as in-depth epidemiological studies and clinical trials of the malaria vaccine candidates Sanaria® PfSPZ Vaccine and PfSPZ-CVac [44, 47, 48] to collect data on safety and efficacy to support vaccine licensure. In 2018, the BIMEP conducted a regimen optimization trial of PfSPZ Vaccine (ClinicalTrials.gov ID: NCT03590340) at the Baney Research Facility that involved 104 healthy Equatoguinean adults male and female, aged 18–35 years [49]. Study volunteers were recruited from Baney district and the city of Malabo, and were enrolled after providing informed consent. Homologous (the strain of *P. falciparum* in PfSPZ Challenge, PfNF54, was the same as in the vaccine) CHMI with 3.2 × 10^3^ PfSPZ was administered to 95 eligible individuals, 6 to 7 weeks after last vaccination and the observation period was conducted in a hotel at the La Paz Hospital beginning eight days after PfSPZ Challenge injection.

### Malaria rapid diagnostic tests

Malaria rapid diagnostic tests were performed with frozen venous whole blood samples anticoagulated with EDTA and stored at − 80 °C. Blood samples were allowed to thaw slowly and equilibrate to ambient temperature for at least 30 min before performing the RDT. The commercially available cRDT [Carestart malaria Pf/PAN (PfHRP2/pLDH) Ag Combo, ACCESSBIO, USA (Lot# MR18F63, expiration: 30th Nov, 2020)] and uRDT [the Alere™ Malaria Ag Pf, Standard Diagnostic Inc., Republic of Korea (Lot# 05LDE001A, expiration: 20th Feb, 2020)] were used throughout the study. Both tests have similar manufacturer instruction workflows, requiring 5 µL blood to be applied to the RDT, followed by addition of the assay buffer and incubation for 20 min. After incubation, diagnosis was determined by the appearance of lines in the test result window. A single control line was considered malaria negative, a line for both control and test were considered malaria positive, and no lines, neither control nor test, was considered an invalid test. Two to three readers were involved in determining each RDT result, the third added when there were discrepancies between the first two readers. The samples were analysed in batches of 10 by a first reader and the developed RDTs were then given to a second reader to confirm the reading of the first reader. Any discrepancy between first and second readers was resolved using a third reader within a period of 20 ± 1 min. Results were recorded in the database as binary figures with zero representing negatives and one representing positives. Five fresh known malaria positive and five fresh known negative samples were used for quality control for the performance of the assay and for training the technicians prior to conducting the study.

### Thick blood smear for quantification of *Plasmodium falciparum*

Two millilitres of fresh whole venous blood from study participants were used for the preparation of TBS. The TBS was prepared by evenly spreading 10 µL of fresh whole venous blood into a 1 cm × 2 cm rectangle. The smears were air dried, stained for 45 min using 4% Giemsa stain and rinsed with buffered water, pH 7.2. The slides were dried and read using a light microscope with a high-power field (immersion oil, 100× objective) of 0.18 mm diameter. 6 × 1 cm passes equivalent to 0.54 µL of blood or 24 × 1 cm passes equivalent to 2.14 µL of blood for symptomatic volunteers were read before a TBS was declared positive or negative. The slides were read by two independent expert microscopists and any discrepancies were resolved by a third microscopist. For Giemsa staining quality control, known positive and negative thin blood smears were included at the beginning of the day and analysed for both parasites and cell staining colour and quality according to a standard operating procedure. Only Giemsa stain that passed the quality control procedures was allowed to be used for the slide staining on that day. Microscopes were maintained on a daily basis.

### Quantification of *P. falciparum* parasite density by polymerase chain reaction (qPCR)

DNA was extracted directly from 180 µL of freshly collected venous whole blood using Quick-DNA Miniprep kits (Zymo Research, Irvine, USA) and eluted with 50 µL of elution buffer as recommended by manufacturer. DNA samples were kept at − 20 °C until analysis using the Bio-Rad CFX96 Real-Time PCR System (Bio-Rad Laboratories, California, USA). The PlasQ assay previously described by Schindler et al. [50] was used for quantification of *Plasmodium spp.* and *P. falciparum* parasites in the venous blood sample. This multiplex assay targets two independent *Plasmodium* genes namely the Pan-*Plasmodium* 18 S rDNA sequence (Pspp18S) and the *P. falciparum*-specific acidic terminal sequence of the *var* genes (PfvarATS). The human Ribonuclease P gene (HsRNaseP) was used as a DNA extraction and qPCR amplification control. All qPCR assays were run in duplicate and both non-template control (molecular grade nuclease-free water) and *P. falciparum* 3D7 DNA were included in each PCR run as negative and positive controls, respectively. For the parasite density estimation, a serial dilution was made according to the 1st WHO International Standard for *P. falciparum* DNA Amplification Technique (NIBSC code: 04/176) to establish a calibration curve with the parasite densities ranging between 0.01 and 10,000 p/µL. The actual parasite density of the tested sample by qPCR was then estimated from the calibration curve’s y-intercept and slope. The lower limit of detection for this qPCR assay was 50 copies/mL. The sample was considered *P. falciparum* positive if each of the two replicates for both PfvarATS and 18 S RNA gene targets had quantitation cycles (Cq) < 40 and Cq < 28 for qPCR amplification control (HsRNaseP). In case of a discrepancy between duplicates, the assay was repeated, with at least two positive replicates out of four considered a positive result. The final results were used for the qPCR-based estimate of parasite density.

### Controlled human malaria infection (CHMI)

From October 2018 to March 2019, 95 healthy Equatoguinean adults underwent CHMI [49]. Prior to CHMI, a full 3-day course of artemether/lumefantrine treatment was given to all volunteers. Eligibility criteria for CHMI were met if volunteers had received a complete regimen of PfSPZ Vaccine and were negative for malaria infection at the time of CHMI. During the ward observation period, volunteers were monitored daily for *P. falciparum* parasitaemia starting on day 8 to detect the parasite early and prevent the development of symptoms. Two millilitres of venous whole blood were collected in EDTA tubes daily on days 8–20 post-infection and transported to the laboratory in cooling boxes (4–8 °C) within 30 min of collection. One mL blood was used for examining malaria parasites positivity and density by TBS and qPCR within 4 h of collection and 1 mL was stored at − 80 °C for 8 months before retrospectively analysing samples using uRDT and cRDT. The standard artemether/lumefantrine treatment was given to subjects once malaria parasites were detected by TBS or on day 28 post CHMI for volunteers who remained negative throughout the post-CHMI follow-up period. Positive TBS results confirmed by qPCR were used as the end-point for initiating participant malaria treatment and termination of ward visits and further diagnostic sample collection. Volunteers diagnosed as malaria positive by TBS during 28 days of CHMI follow-up were considered eligible for participation in this malaria diagnostics study.

### Sampling and statistical analyses

The aim of this study was to demonstrate differences in performance sensitivity between TBS, cRDT, and uRDT methods in detecting low density malaria infection, using qPCR as the gold standard for sensitivity. To obtain sufficient samples, Epi Info 7 software was used to calculate the sample size assuming the following parameters: minimum sensitivity of 90% for a reference method (qPCR) and 80% for cRDT, a 95% confidence and 90% power to detect a maximum sensitivity difference of 10%. cRDT was used to determine sample size due to its wider application in the field [51]. This gave a minimum required sample size of 267 samples. Sensitivity of the various tests was compared using Fisher’s exact test.

Participating individuals were to be observed from day 8 after challenge until the day of first positivity by TBS. Tests were not performed on samples collected after TBS diagnosis since volunteers were treated and positive results could still occur for RDTs and qPCR due to residual parasite material, which would confound results. Out of the eligible volunteers, a subset of 24 individuals were to be randomly selected to meet sample size requirements while maintaining the distribution of parasite densities observed using qPCR. Considering the low sensitivity of most cRDTs at 100 p/µL, a stratified random sampling method was selected, whereby samples with parasite density > 100 p/µL and < 100 p/µL as detected by qPCR were put into two different strata. Using Microsoft Excel (2016), simple random sampling was performed within each stratum to obtain a total of 24 individuals.

To assess the distributions of the complete set, sampled and unsampled subsets were examined to ensure that they presented similar structure. Sampled individuals provided a total of 279 observed individual sample time points.

For this analysis, a multiplex qPCR targeting Pspp18S and PfvarATS was designated as the reference for detection of infection against which TBS, uRDT and cRDT positivity could be compared. A two-tailed Fishers exact test was used to determine the significant differences between the sensitivities of the various diagnostic tests. In this study, all samples were included in the sensitivity analysis of diagnostics. Only positive samples by either TBS, uRDT and cRDT were included in the analyses of the overall geometric mean (geomean) of parasite density of positive results and the geomean of parasite density at time of first detection (prepatent period). If a TBS, uRDT or cRDT test was negative, the respective sample point was deemed not applicable for the geomean parasite density and time to first detection analysis. TBS and qPCR both provided density measurements but TBS was considered to be more reliable as it did not involve conversion from gene copy number using a reference standard.

Results were recorded by trained and qualified laboratory staff on case report forms (CRFs) during the CHMI ward observation period and later entered onto an Excel spreadsheet (Microsoft, Office 2019 Ver 16). All samples were assigned a sample specific number that was linked to each volunteer ID. No personal information was recorded on laboratory CRFs and for the laboratory staff the connection of each sample with the corresponding donor volunteer was not possible. Retrospective RDT results were recorded on the same Excel spreadsheet. The geomean and geomean confidence intervals of parasite densities were calculated using R 4.0.1. Sensitivity and 95% confidence intervals for all diagnostic methods were calculated in R 4.0.1 using the epiR package [52].

## Results

### Overview

A total of 48 volunteers were diagnosed positive for malaria by reference method (qPCR), qualifying them for inclusion in the malaria diagnostic comparison. Individuals had an average of 12 time points of observation-days (range 8 to 17), with each day-test-record representing an independent observation since each was obtained from a newly collected whole blood sample. Out of the eligible volunteers, 24 (50%) individuals were randomly selected while maintaining the distribution of parasite densities observed using qPCR. The subset of selected samples was evaluated against unselected samples by parasite density distribution and variance and the selected subset was determined to be an appropriate representation (Additional file 1: Figs. S1–S3).

**Fig. 1:**
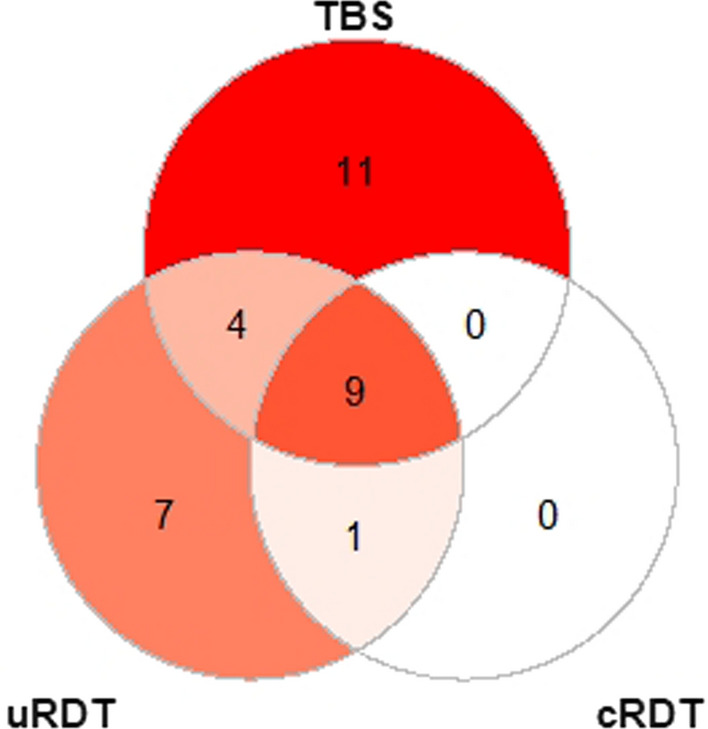
Similarities and discrepancies in detection of *P. falciparum* cases by different diagnostic methods. Venn-diagram showing distribution of positive results according to the diagnostic test used with qPCR as the reference method. All samples were sorted by thick blood smear (TBS), ultrasensitive rapid diagnostic test (uRDT), and conventional rapid diagnostic test (cRDT). All cases were low parasite density *P. falciparum* infections that occurred during CHMI

A total of 279 samples were collected from the 24 selected study participants; 123 and 156 samples were positive and negative for *P. falciparum* infection by qPCR, respectively. All 156 samples negative for *P. falciparum* by qPCR were also negative by TBS, uRDT and cRDT demonstrating 100% specificity for these tests. In total, 24 of 123 positive samples were detected by TBS, 21 by uRDT and 10 by cRDT, providing sensitivities of 19.5% (95% CI 13.1–27.8%), 17.1% (95% CI 11.1–25.1%), and 8.1% (95% CI 4.2–14.8%), respectively. qPCR detected more positives than any of the other tests (p < 0.001) and TBS and uRDT were both more sensitive than cRDT (TBS vs. cRDT, p = 0.015 by Fishers Exact two-tailed; uRDT vs. cRDT, p = 0.053). TBS detected 61.9% (13/21) of uRDT positive infections, while uRDT detected 54.2% (13/24) of TBS positive infections. The uRDT detected 100% (10/10) of cRDT positive infections while TBS detected 90% (9/10). The cRDT detected 47.6% (10/21) of uRDT positive infections and 37.5% (9/24) of TBS positive infections. The summary of the findings are depicted in a Venn-diagram (Fig. 1).

Parasite density by qPCR was calculated using a standard curve generated using a WHO reference sample that related copy number to density, and ranged from 0.14 to 603.8 p/µL, with a geomean of 2.57 p/µL. Using this scale TBS detected parasites in the range of 5.9–603.8 p/µL (geomean 97.6 p/µL) and uRDT detected parasites in the range of 0.8–603.8 p/µL (geomean 103.4 p/µL), compared to cRDT, which detected parasites in the range of 2.6–603.8 p/µL (geomean = 149.4 p/µL) (Table 1).

**Table 1:** Overall geomean and sensitivity of TBS, uRDT, and cRDT compared to the qPCR method

Diagnostic test	TBS (+)	TBS (−)	uRDT (+)	uRDT (−)	cRDT (+)	cRDT (−)	Total
PCR Pf (+)	24	99	21	102	10	113	123
PCR Pf (−)	0	156	0	156	0	156	156
Total	24	255	21	258	10	269	279
Range of positive samples (p/µL by qPCR)	5.9–603.8		0.8–603.8		2.6–603.8		
Geomean of positive samples (p/µL by qPCR)	97.6		103.4		149.4		
Sensitivity (%)	19.5% (13.1–27.8)		17.1% (11.1–25.1)		8.1% (4.2–14.8)		

Number of positive and negative samples, overall geomean and sensitivity of TBS, uRDT, and cRDT compared to the reference qPCR method. All cases were low parasite density infections that occurred during CHMI. Parasite densities of positive samples by qPCR ranged from 0.14–603.84 p/µL

Parasite density by TBS ranged from 3.7 to 201.8 p/µL (geomean 12.81 p/µL). Using this scale, uRDT detected parasites in the range of 3.7–201.8 p/µL (geomean 20.6 p/µL), compared to cRDT, which detected parasites in the range of 5.6–201.8 p/µL (geomean = 30.2 p/µL) (Table 2).

**Table 2:** Overall geomean and sensitivity of uRDT and cRDT compared to the TBS method

Diagnostic test	uRDT (+)	uRDT (−)	cRDT (+)	cRDT (−)	Total
TBS (+)	13	11	9	15	24
TBS (−)	8	247	1	254	255
Total	21	258	10	269	279
Geomean of positive samples (p/µL by TBS)	20.6		30.2		
Sensitivity (%)	54.2% (33.2–73.8)		37.5% (19.6–59.2)		

Overall geomean, sensitivity and number of positive and negative samples of uRDT, and cRDT compared to TBS. All cases occurred during CHMI. Parasite densities of positive samples by TBS ranged from 3.7 to 201.8 p/µL

Examining just infections positive by both qPCR and TBS, the geomean ratio established from parasite density of qPCR and TBS (qPCR/TBS) was 7.62 p/µL (Table 3). PCR detects gene copy number in a specimen and each *P. falciparum* genome has at least 5–8 copies [53], and in addition qPCR can detect free DNA in a specimens. These variables make it difficult to calculate parasite density accurately using qPCR, even when using a standard curve to convert copy numbers to density based on a WHO reference sample. TBS detects parasites, regardless of how many genes and nuclei are present, but is hindered by the possible loss of significant numbers of parasites during processing [54]. All these factors were suspected to have contributed to the higher densities found using qPCR compared to TBS.

**Table 3:** Overall geomean, ranges and the ratio of parasite density established from qPCR and TBS

	qPCR (Pf/µL)	TBS (Pf/µL)	Ratio qPCR/TBS
GeoMean	97.57	12.81	7.62
Range	[5.86–603.84]	[3.70–201.80]	[0.07–53.84]

Comparison of geomean, ranges and the ratio of parasite density established from qPCR and TBS using paired samples in which both tests were positive. All cases were low parasite density infections that occurred during CHMI. The Ratio was determined by dividing the geomean of qPCR by geomean of TBS (qPCR/TBS). N = 24

Next, the diagnostic test sensitivities of TBS, uRDT and cRDT were stratified by ranges of parasite density of qPCR (Table 4). None of the three diagnostic tests detected *P. falciparum* infections below 1 p/µL as determined by qPCR. For TBS, the lowest parasite density detected as quantified by qPCR was 5.86 p/µL and as quantified by TBS was 3.7 p/µL. At parasite densities of 1–50 p/µL, uRDT and TBS appeared roughly equally sensitive and both appeared more sensitive than cRDT. At parasite densities of 51–100 p/µL by qPCR, uRDT appeared more sensitive (71% [95% CI 29–96%]) compared to TBS and cRDT with, 29% (4–71%) and 29% (4–71%) sensitivity, respectively. However, numbers were small and differences in sensitivity amongst the tests across the density categories were not statistically significant.

**Table 4:** Comparison of TBS, uRDT and cRDT sensitivity stratified by parasite density (p/µL) as determined by qPCR

Group density (p/µL)	# samples qPCR (+) (reference)	TBS (+)	TBS sensitivity (95% CI)	uRDT	uRDT sensitivity (95% CI)	cRDT	cRDT sensitivity (95% CI)
< 1	37	0	–	0	–	0	–
1–10	38	1	3% (0–14)	1	3% (0–14)	1	3% (0–14)
11–50	25	7	28% (12–49)	4	16% (5–36)	0	–
51–100	7	2	29% (4–71)	5	71% (29–96)	2	29% (4–71)
> 100	16	14	88% (62–98)	11	69% (41–89)	7	44% (20–70)

Number of samples and sensitivity of TBS, uRDT and cRDT stratified by parasite density (p/µL). All cases were low parasite density samples that occurred during CHMI follow-up and were 100% specific compared to qPCR

Finally, the uRDT and cRDT diagnostic test sensitivities were stratified by ranges of parasite density measured by TBS (Table 5). At parasite densities range between 1 and 50 p/µL, uRDT had higher sensitivity compared to cRDT; 33% (95% CI 12–62) for 1–10 p/µL and 100% (95% CI 48–100) at 11–50 p/µL compared to 13% (95% CI 2–40) for 1–10 p/µL and 80% (95% CI 28–99) at 11–50 p/µL respectively. Above 50 p/µL, both uRDT and cRDT had the same sensitivity but again numbers were too small to allow a meaningful comparison.

**Table 5:** Comparison of uRDT and cRDT sensitivity stratified by parasite density (p/µL) as determined by TBS

Group density (p/µL)	# samples TBS (+) (reference)	uRDT (+)	uRDT sensitivity (95% CI)	cRDT (+)	cRDT sensitivity (95% CI)
< 1	0	0	–	0	–
1–10	15	5	33% (12–62)	2	13% (2–40)
11–50	5	5	100% (48–100)	4	80% (28–99)
51–100	2	2	100% (16–100)	2	100% (16–100)
> 100	2	1	50% (1–99)	1	50% (1–99)

Number of samples and sensitivity of uRDT and cRDT stratified by parasite density (p/µL). Diagnostic methods are compared to TBS as reference and all cases were low parasite density samples that occurred during CHMI follow-up

**Fig. 2:**
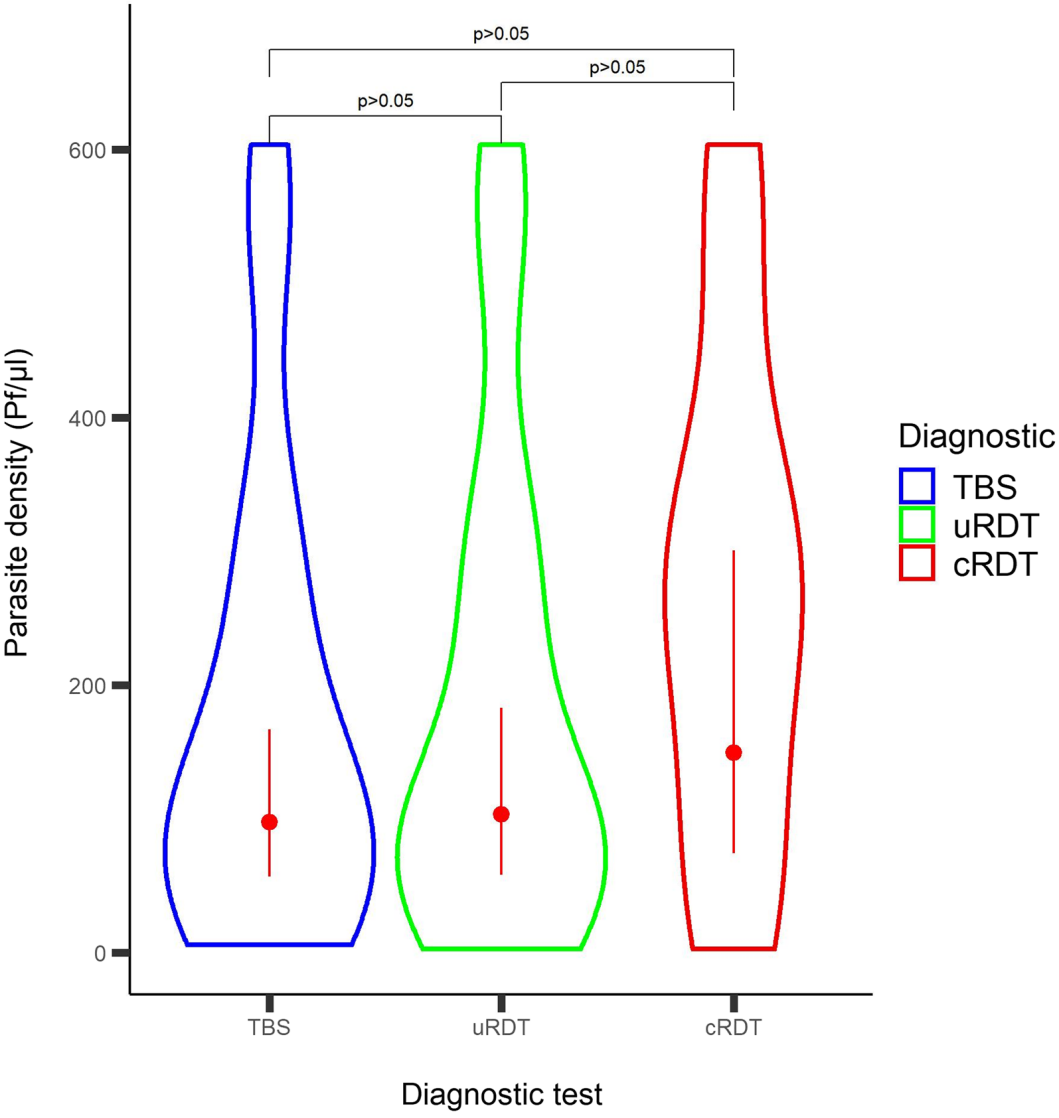
Distribution of parasite density by qPCR of all malaria positive samples by TBS, uRDT, and cRDT. Red dots represent the geomean and error bars represent the 95% confidence intervals of each respective diagnostic test. Significance values were calculated using two-tailed Wilcoxon-test

Finally, the range and distribution of parasite densities of samples determined to be positive by qPCR (n = 123), by TBS (n = 24), by uRDT (n = 21) and by cRDT (n = 10) were examined over the follow up period for the 24 volunteers who were TBS positive. TBS and uRDT recorded a trend for lower geomean parasite densities detected compared to cRDT, which did not reach statistical significance [p = 0.19 and p = 0.26, respectively] (Fig. 2).

### Time to first detection

**Fig. 3:**
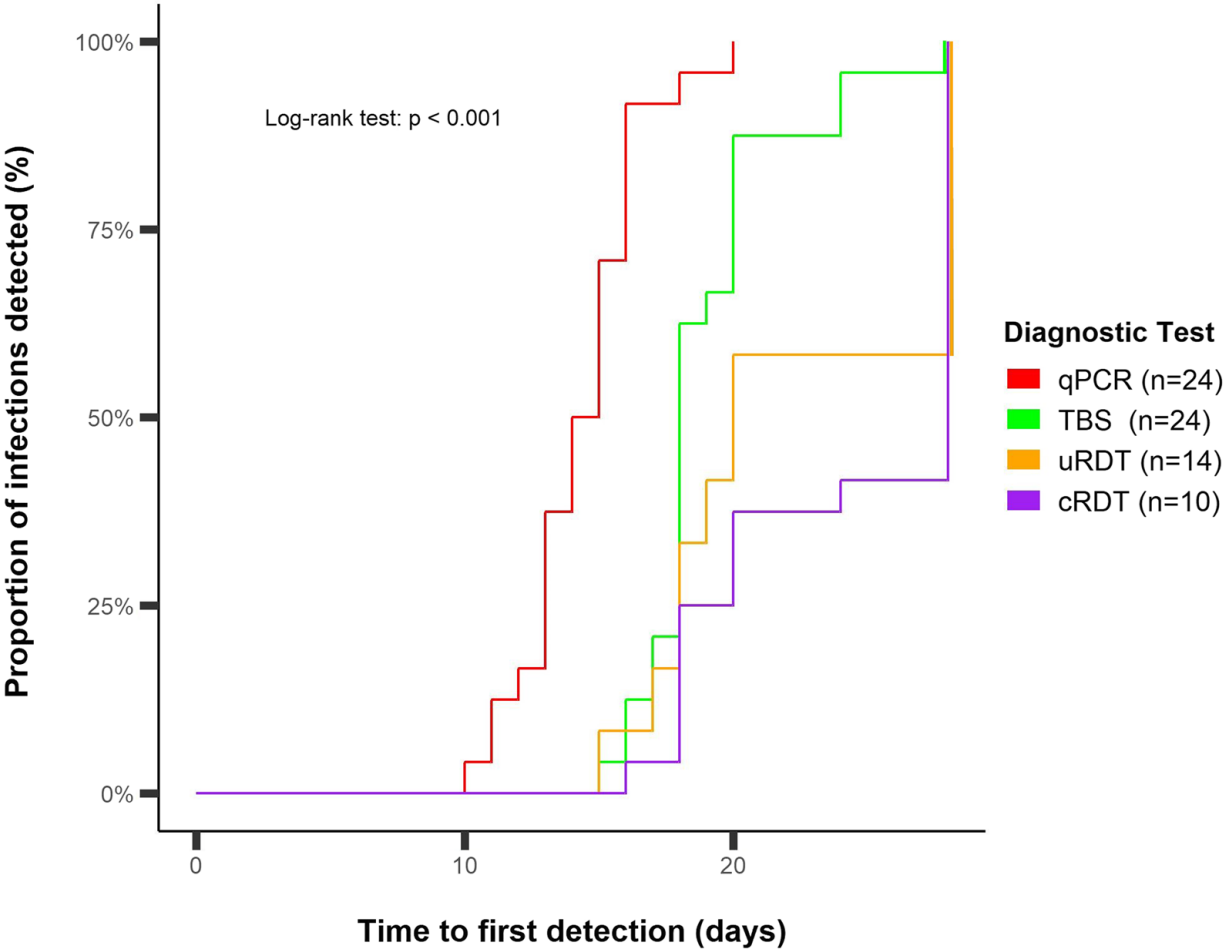
Comparison of time to detection of parasites. Kaplan–Meier plot of the number of infections detected by time since CHMI qPCR (N = 24), TBS (N = 24), uRDT (N = 14) and cRDT (N = 10). All cases were low parasite density Pf infections that occurred during CHMI. P-value < 0.001 calculated using the log-rank test

To investigate the efficiency of cRDT and uRDT to detect asexual blood stage parasites during CHMI follow-up, the median times to first detection of parasites (in days) by qPCR, TBS, uRDT and cRDT were compared. There was no evidence to support differences in prepatent period when using TBS, uRDT and cRDT since these methods all reported a median of 18.0 days to first parasite detection. The median days to detection of asexual blood stage parasitaemia by qPCR was 14.5, 3.5 days earlier than TBS, uRDT and cRDT (p < 0.001 log-rank test) (Fig. 3).

## Discussion

As progress against malaria is made, asymptomatic infections at lower parasite densities become a significant challenge for malaria control and elimination efforts due to their contribution to ongoing transmission [3, 4, 55, 56]. Mass testing of a population with treatment of those found positive is one approach to address this problem [57]. However, it is difficult to diagnose low density parasitaemias and the most sensitive and, therefore, the best method, qPCR, is expensive, requires special laboratories and skilled personnel. Thus, the development of an inexpensive rapid test with equivalent sensitivity would be of great benefit, especially as conventional rapid diagnostic tests (cRDTs) are significantly less sensitive. Other applications could also benefit from a simple, rapid test that is more sensitive than cRDTs, such as detection of parasitaemia following sporozoite or blood stage CHMI, both important procedures for evaluating vaccine and drug efficacy [44, 45, 58–61] or for exploring innate and acquired immunity [62–66]. PfSPZ CHMI in particular is now gaining attention by investigators, since it can now be used by any clinical centre without the need for infectious mosquitoes [67–69]. In CHMI, detection of low parasite densities is useful because it allows the identification and treatment of positive study subjects earlier in the course of their parasitaemia thereby preventing or ameliorating clinical manifestations. Therefore, on many fronts, there is a need to develop simpler, highly sensitive methods to diagnose low parasite densities that could augment the success of mass testing and treatment, promote epidemiological studies and simplify and lessen the costs associated with CHMI.

cRDTs have been a tremendous boon to diagnosing clinical malaria, where parasite densities are relatively high and the tests adequately sensitive. An uRDT has recently been developed, and might extend the usefulness of RDTs particularly in low to moderate transmission areas, in pre-elimination settings, and in experimental uses such as CHMI follow-up. For example, it has been reported that the uRDT is significantly more sensitive than cRDTs and TBS, detecting PfHRP2 at parasite densities as low as 0.1–1.0 p/µL in culture-derived samples [7, 23]. For this reason, the current study tested the uRDT and a cRDT during follow-up in a CHMI trial, and compared their sensitivities to those of TBS, using qPCR as the reference standard.

This study indeed found that both TBS and the uRDT were more sensitive than the cRDT. However, the data demonstrated that in samples from malaria-experienced subjects undergoing CHMI with parasites that contained PfHRP2, the sensitivity of uRDT (17.1%) was about threefold lower than that reported for pretreatment specimens from an IBSM study (47%) and in samples from a low transmission setting (44%), and fivefold lower than that reported in samples from a high transmission setting (84%) [23]. None of 37 specimens less than 1.0 p/µL by qPCR were identified. In 15 specimens that had 1–10 p/µL by TBS, uRDT identified 5 (33%), and cRDT 2 (13%). In 5 specimens with 11–50 parasites/µL by TBS, uRDT identified all 5 as positive (100%) and cRDT identified 4 (80%). Overall, uRDT and TBS gave similar results, and both tests were more sensitive than cRDTs in a setting of CHMI with PfHRP2-containing parasites.

Currently, CHMI requires highly trained clinical and laboratory staff including expert microscopists. Considering the comparable outcomes of TBS and uRDT in this study, uRDT could be considered to replace TBS microscopy, especially in settings with inexperienced microscopists. However, parasite density estimation using qPCR has become a standard method utilized in many malaria studies [70, 71] and has been particularly useful in CHMI follow-up where it can detect parasitaemia earlier than TBS and allow treatment before signs and symptoms of clinical malaria develop [72–74]. Thus, uRDT would need to show advantages over TBS in the early identification of positive study subjects, as TBS itself is now being supplanted by qPCR. As expected, the study showed a significant difference in prepatent period amongst qPCR on the one hand and TBS, uRDT and cRDT on the other, confirming that qPCR is the most sensitive diagnostic method. The study further demonstrated that the median time to first malaria parasite detection by qPCR was 3.5 days earlier (14.5 days) compared to the other tests, and also that TBS, uRDT and cRDT were substantially equivalent to each other, each providing a prepatent period of 18 days. Similar to these findings, the CHMI studies conducted in semi-immune participants [44, 58, 73, 75] and in malaria naïve participants [76] have reported comparable prepatent periods using qPCR as the reference method. The fact that in this setting of progressively rising parasitaemias, the prepatent periods calculated by TBS, uRDT and cRDT were similar even though positive samples diagnosed by uRDT had a lower overall geomean of parasite density by qPCR than did TBS, suggests that uRDT may not have any particular advantage over TBS other than reduced costs and easier performance, or even over cRDT, as in this study the day of treatment would not have been affected had it been cRDT- rather than TBS-based. It would be expected that the same relative detection abilities would hold for blood stage CHMI, although this was not evaluated in this study, and Das et al. reported that uRDT detected parasitaemia 1.5 days earlier than cRDT in this setting [23].

In a field setting, the greater sensitivity of the uRDT over cRDTs could allow the detection of more asymptomatic carriers. This question was not directly examined in this CHMI-based study. However, the results showed that despite the uRDT being hailed as a significant improvement in malaria diagnostics, leading to increased sensitivity and specificity, satisfactory RDT performance for parasite density infections < 10 p/µL remains elusive. Field studies of mass testing and treatment are needed to further explore the potential contribution of the uRDT in identifying and treating asymptomatic carriers with low parasite densities contributing to ongoing transmission.

One important consideration for evaluating RDTs based on the detection of PfHRP2 is the increase in prevalence of parasites carrying PfHRP2 deletions, not only in Southeast Asia [77], but within the study area as well [12]. In 2018, approximately 65% of all suspected malaria cases in public health facilities in sub-Saharan African were tested with RDTs (~ 150 million cases) [1]. During CHMI, a standardized infectious PfSPZ dose of PfSPZ Challenge (NF54) was used, a parasite that expresses PfHRP2 to initiate the infection. Conducting a similar study in hospitals and field environment with important confounders, such as a deleted *Pfhrp2* gene, would likely have had different results.

## Limitations

Anticoagulated (EDTA) fresh whole blood was used for to prepare samples for qPCR and TBS assessments. Anticoagulated (EDTA) cryopreserved (temperature of − 80 °C) whole blood held for 8 months and thawed was used to prepare samples for uRDT and cRDT. It is possible, but unlikely that HRP2 degraded during storage. Whole blood samples were temperature monitored during storage. When proper procedures are followed for long-term storage of whole blood, the quality of DNA, RNA or HRP2 is not compromised [7, 78]. Considering the strict temperature monitoring in this study and the fact that samples were only thawed once for processing, the difference in quality of samples over time is unlikely to have been different.

Another limitation is the discrepancy in parasite densities estimated by qPCR and TBS. qPCR may have overestimated parasite density due to variable numbers of copies of the amplification target and the persistence of nucleic acid from non-viable parasites [79], and TBS may have underestimated parasite density due to the loss of parasites during processing.

Because the research subjects were semi-immune, they may have had variable levels of anti-HRP2 or anti-LDH antibodies, which could have affected results [80, 81]. Some may also have had ongoing infections at the time of clearance with artemether/lumefantrine prior to CHMI. Although the current study did not measure the level of HRP2 in the participants before and after CHMI, none of the individuals were RDT positive between days 8 and 14 after CHMI. Therefore, it is likely that this factor did not affect the current performance comparison.

This study was designed to evaluate the performance of malaria diagnostic tests in independent samples and was analysed accordingly, even though several samples were collected from each individual post CHMI. This was based on the reasoning that, since each test was measuring a different parameter (DNA for qPCR, whole parasites for TBS, HRP2 for uRDT, HRP2/LDH for cRDT) and these parameters would vary independently from day to day due to the presence of multiple clones of NF54 parasites released from individual hepatocytes over several days each with its own asynchronous 48 h reproductive/sequestration cycle, it would be difficult to propose a biological metric characterizing an individual that could introduce bias or similarities in observations. Nevertheless, such a bias or similarities could exist and might have affected the data.

## Conclusions

TBS has been the classical approach to malaria diagnosis for clinical use, malaria control programs, research studies such as CHMI and field epidemiology. TBS can distinguish the five malaria species that infect humans, which cannot yet be achieved by using RDTs or a single reaction qPCR, and provides a reasonable estimate of parasite density. TBS, however, requires laboratories that support and maintain microscopes, staining solutions and human resources with the requisite microscopy skills. qPCR, with much greater sensitivity, is now supplanting TBS for many applications such as detection of parasitaemia following CHMI, but also requires a high level of laboratory capability and involves higher costs than TBS. cRDTs have, therefore, been a welcome addition to malaria diagnostics and in many places have supplanted TBS for the clinical diagnosis of malaria, where parasite densities are high, but have not been useful for applications requiring greater sensitivity. This study compared qPCR, TBS, a cRDT to a new uRDT advertised as rivaling qPCR in sensitivity, to assess its value for detection of parasitaemia following PfSPZ CHMI, an application where early diagnosis and treatment is important to reduce the severity of adverse events. The major conclusions were that for this specific application, while the uRDT was better than the cRDT, and approached TBS in sensitivity, it did not close the gap with respect to qPCR, and thus could be considered for replacing TBS only in studies unable to use qPCR or TBS due to resource limitations. The added value of the uRDT in field studies, particularly in mass testing and treatment, requires further study.

## Supplementary Information

**Additional file 1: Figure S1.** Trend ofparasite density over time between individuals in selected and unselected groups.Trend of parasite density over time between selected and unselectedparticipants. Parasite density was determined by quantitative polymerase chainreaction assays (qPCR). **Figure S2.** Overall distribution of parasite densityamong groups of selected and unselected participants. Scatter plots of parasitedensity measured by quantitative polymerase reaction assays (qPCR) betweenselected and unselected data points. **Figure S3.** Distribution of parasitedensity by individuals participants in selected and unselected groups. Barplotsof parasite density as measured by quantitative polymerase reaction assays(qPCR) between selected vs. unselected individual volunteers.

## Data Availability

The datasets used and/or analysis during the current study are available from the corresponding author on reasonable request.

## Abbreviations

cRDT: Conventional rapid diagnostic test
PfHRP2: Histidine-rich protein 2
PAN: Pan-malaria antigen
TBS: Thick blood smear
p/µL: Parasites per microlitre
qPCR: Quantitative polymerase chain reaction
uRDT: Ultra-sensitive rapid diagnostic test
CHMI: Controlled human malaria infection
DVI: Direct venous inoculation
BIMEP: Bioko Island malaria Elimination Project
CRFs: Clinical report forms
