# Supplementary figures and images for: Diagnostic performance and comparison of ultrasensitive and conventional rapid diagnostic test, thick blood smear and quantitative PCR for detection of low-density Plasmodium falciparum infections during a controlled human malaria infection study in Equatorial Guinea
Source: Malar J. 2022 Mar 24;21:99. doi: 10.1186/s12936-022-04103-y (PMC8943516; doi:10.1186/s12936-022-04103-y)

**Additional Figures**

**Figure S1**


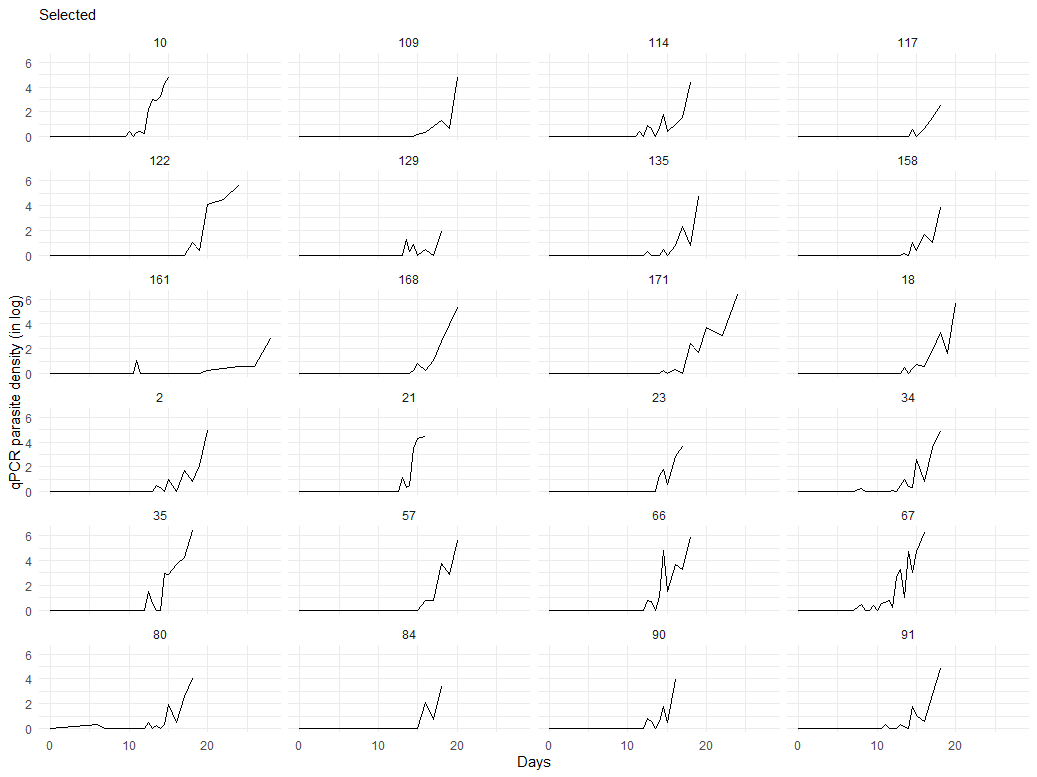

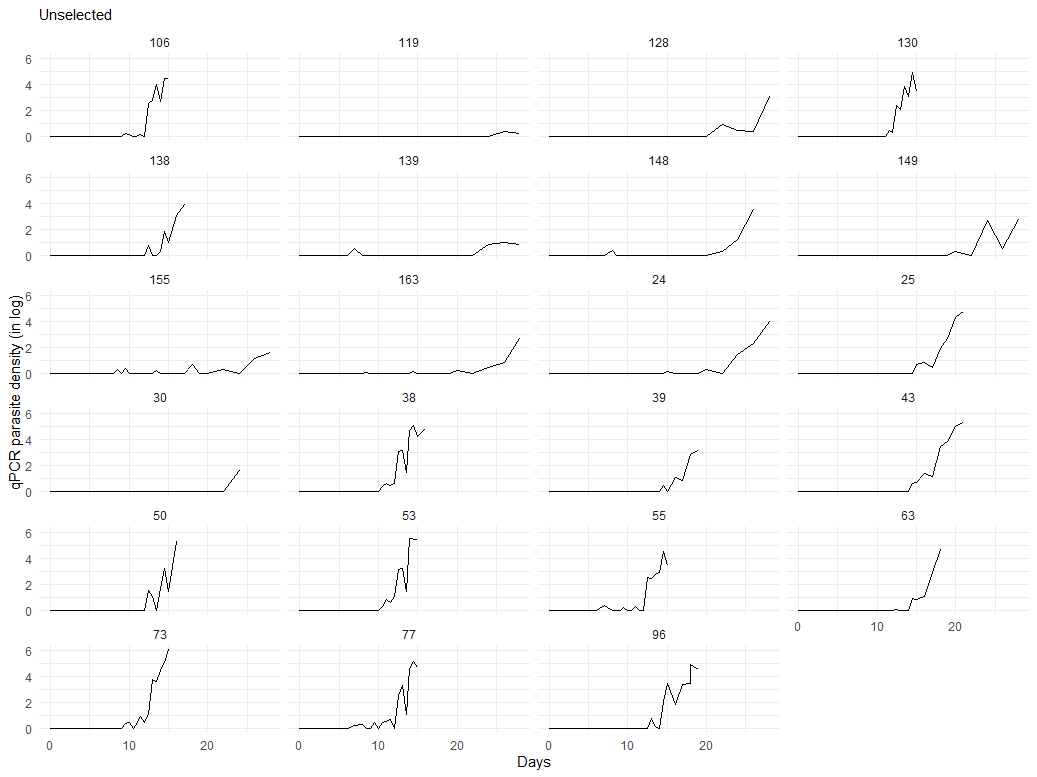


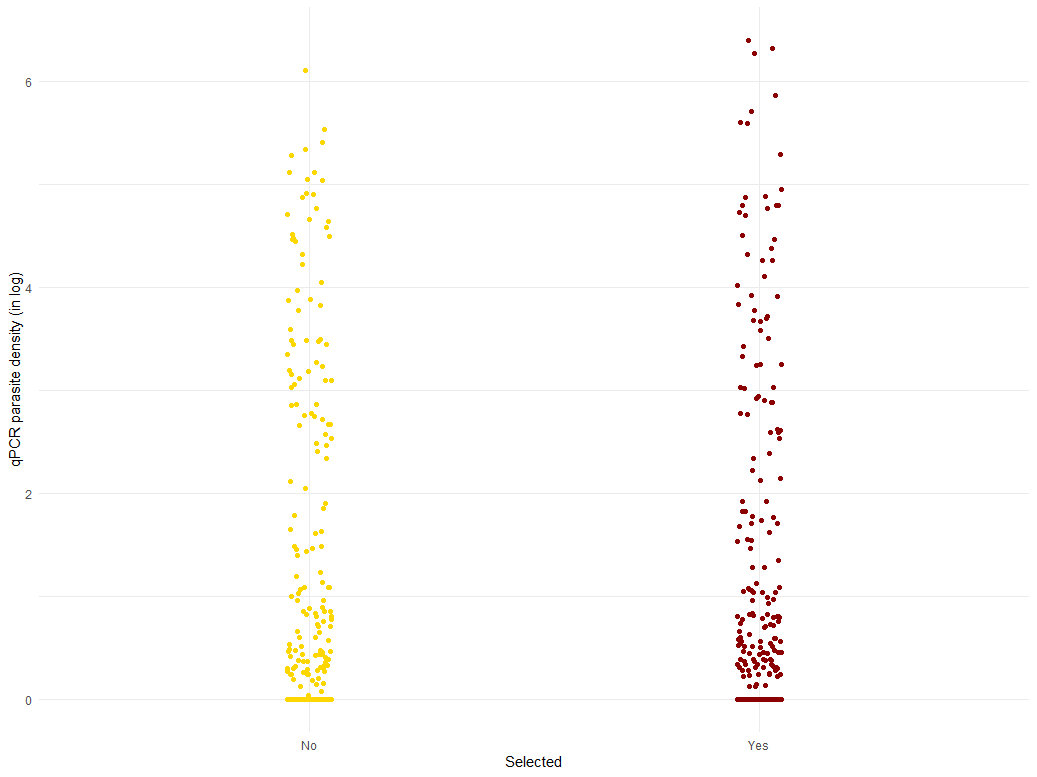
**Figure S2**

**Figure S3**


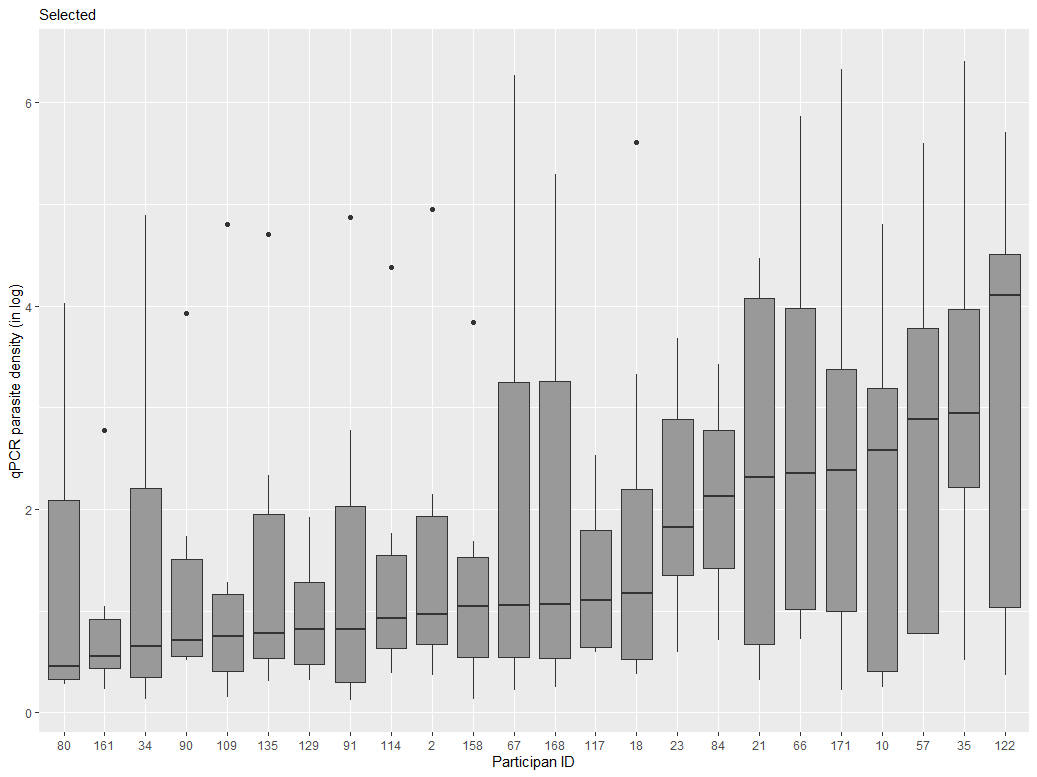

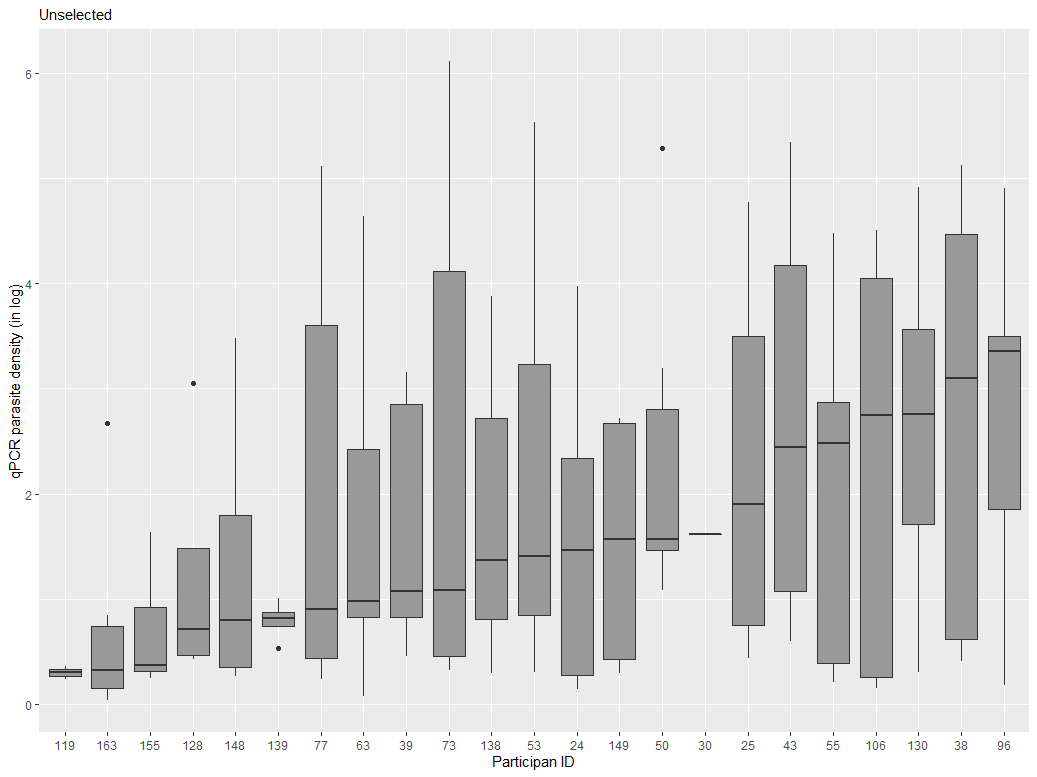

Supplement: Supplementary file 1 — Additional file 1: Figure S1. Trend ofparasite density over time between individuals in selected and unselected groups.Trend of parasite density over time between selected and unselectedparticipants. Parasite density was determined by quantitative polymerase chainreaction assays (qPCR). Figure S2. Overall distribution of parasite densityamong groups of selected and unselected participants. Scatter plots of parasitedensity measured by quantitative polymerase reaction assays (qPCR) betweenselected and unselected data points. Figure S3. Distribution of parasitedensity by individuals participants in selected and unselected groups. Barplotsof parasite density as measured by quantitative polymerase reaction assays(qPCR) between selected vs. unselected individual volunteers. [file 12936_2022_4103_MOESM1_ESM.docx]
